# PARP1 inhibition enhances reactive oxygen species on gut microbiota

**DOI:** 10.1002/jcp.30861

**Published:** 2022-08-22

**Authors:** Yixiao Zhuang, Hui Wang, Jiyang Ding, Xinyi Zhang, Xiaoyu Liu, Shuai Zhang, Xiaorui Xing, Keneilwe Kenny Kaudimba, Muyang He, Shuang Zhang, Shanshan Guo, Xingxing Kong, Li Jin, Tiemin Liu

**Affiliations:** ^1^ State Key Laboratory of Genetic Engineering, School of Life Sciences Fudan University Shanghai China; ^2^ Shanghai Key Laboratory of Metabolic Remodeling and Health, Institute of Metabolism & Integrative Biology Fudan University Shanghai China; ^3^ College of Life Sciences Inner Mongolia University Hohhot Inner Mongolia China; ^4^ School of Kinesiology, Key Laboratory of Exercise and Health Sciences of Ministry of Education Shanghai University of Sport Shanghai China; ^5^ Shanghai Medical College Fudan University Shanghai China; ^6^ Human Phenome Institute Fudan University Shanghai China

**Keywords:** gut microbiota, NF‐κB, poly(ADP‐ribose) polymerase 1, ROS, UV

## Abstract

Poly(ADP‐ribose) polymerase 1 (PARP1) plays a key role in genome stability by modulating DNA‐damage responses. Activated by DNA interruptions through ultraviolet (UV) exposure, PARylation is synthesized by PARP1 and serves as a survival mechanism for cancer and metabolic diseases. Several strategies including ROS and antimicrobial peptides (AMPs) function in host defenses, while the targeted tissue and mechanism under DNA damage are unknown. Here, we show that DNA damage induces responses specifically in the gut tissue. The knockdown of PARP1 reduces the activation of PARylation. Parp1 knockdown under DNA damage results in over‐accumulated ROS and secretion of AMPs through the regulation of Relish, a subunit of nuclear factor‐κB (NF‐κB). Double‐knockdown of Parp1 and Relish specifically in the gut inhibits AMP secretion. In conclusion, the host defense is achieved through ROS accumulation rather than the AMPs under DNA damage. In contrast, the knockdown of PARP1 exacerbates ROS accumulation to a harmful level. Under this circumstance, NF‐κb targeted AMP secretion is provoked for host defense. Microbiome and functional analysis provide evidence for the hazard of DNA damage and show variations in the metabolic pathways following Parp1 inhibition. Our findings suggest the notion that PARP1 inhibition contributes to ROS accumulation under DNA damage and its role in NF‐κb activation for host defense.

## Graphical abstract

DNA damage activates ROS and inhibited AMPs specifically in the gut tissue. The elimination of PARP1 exacerbates ROS accumulation and changes the host defense balance from ROS to IMD/Relish pathway AMPs under DNA damage.

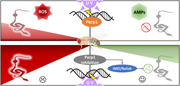

## 1 INTRODUCTION

PARP1, an important member of the poly(ADP‐ribose) polymerase (PARP) family (Jacobson et al., 2001), is activated by DNA interruptions as a DNA repair gene (Gibson & Kraus, 2012). Poly(ADP‐ribosyl)ation (PARylation), synthesized by PARP1 (Jacobson et al., 2001), is a survival mechanism for ultraviolet (UV)‐induced DNA damages (Lakatos et al., 2013). PARP1 inhibitors, such as Olaparib, Rucaparib, Niraparib, and Talazoparib, have been approved by the FDA to treat cancers (Faraoni & Graziani, 2018). However, the mechanism for PARP1 in the ROS and host defense under DNA damage is largely unknown.

DNA damages, generated by environmental stress of UV (O'Donovan et al., 2005), activate the DNA repair genes and eventually result in deleterious effects on energy expenditure, with the generation of ROS which is a byproduct of mitochondrial energy metabolism (Finkel & Holbrook, 2000). Studies have focused on the whole body under DNA damage hazards while the reaction of gut has been masked by other tissues (Buchon et al., 2009).

Intestinal homeostasis is achieved through several strategies (Guo et al., 2014; Li et al., 2020): reactive oxygen species (ROS), the production of antimicrobial peptides (AMPs), melanization reaction, and phagocytosis. ROS, generated by NADPH enzymes Duox and Nox (Ha et al., 2009; Ha, Oh, Bae, et al., 2005), is a barrier of defense while the activation of AMPs, melanization reaction, and phagocytosis play complementary roles to microbicidal oxidants for defense (Ryu et al., 2006). However, the balance between ROS and other barriers of host defense under DNA damage is unclear.

Our study shows that the gut tissue responds to DNA damage. Under DNA damage, the host defense is achieved through ROS accumulation rather than the AMPs. In contrast, the knockdown of PARP1 exacerbates ROS accumulation to a harmful level. Under this circumstance, nuclear factor‐κB (NF‐κb) targeted AMP secretion is provoked for host defense. Microbiome and functional analysis provide evidence for the hazard of DNA damage and show variations in the metabolic pathways following Parp1 inhibition.

## 2 MATERIALS AND METHODS

### 2.1 Fly strains

The following fly strain was obtained from Bloomington Stock Center: *UAS‐Relish* ^RNAi^ (RRID: BDSC_33661). *UAS‐CG40411 Parp1* ^RNAi^ (II) (TH201500671.S) was obtained from Tsinghua Fly Center. *UAS‐Luciferase* ^RNAi^ (RRID: BDSC_35788), *esg‐gal4* ^*ts*^ (RRID: BDSC_92832), *tub‐gal4* ^*ts*^, and wild‐type fly *w1118* (RRID: BDSC_3605) were gifted from Xinhua Lin (Fudan University). *Parp1* ^RNAi^ *‐Relish* ^RNAi^ double transgenic fly line was generated by standard recombination genetic crosses of *Parp1* ^RNAi^ and *Relish* ^RNAi^ lines. For details, please see Supporting Information.

### 2.2 Oxidative stress resistance assay

Flies were fed with 300 μl of 5% H_2_O_2_ solution. The activity data were extracted at 1 h bin (Wang et al., 2020). Over 100 flies were used for each line (Belyi et al., 2020). For details, please see the Supporting Information.

### 2.3 DNA damage by UV radiation

DNA damage was generated by UV radiation and 20–25 flies were housed in quartz glass vail, through which UV irradiation was able to transmit (Y. L. Liu et al., 2022). Flies were exposed to 0.6 mW/cm^2^ UV for 2 h. For details, please see Supporting Information.

### 2.4 Protein extraction and western blot

The protein extraction and western blot were carried out as described in the previous study (Zhu et al., 2020). Each sample with 25–30 flies (50 ugs) was loaded onto sodium dodecyl sulfate‐polyacrylamide gel electrophoresis under standard conditions, transferred, and probed with the antibodies. Antibodies are PARP (Cell Signaling Technology, Cat#9532T, RRID: AB_659884), β‐Actin (Abclonal, Cat#AC004, RRID: AB_2737399), and PAR (Trevigen, Cat#4335‐MC, RRID: AB_2572318). For details, please see Supporting Information.

### 2.5 Quantitative real‐time PCR

The quantitative real‐time PCR was carried out as described in the previous study (Kong et al., 2018; Xiao et al., 2021). The standardized RP49 mRNA was used as the invariant control. Supporting Information: Table S1 lists the sequence of used primers in the study. For details, please see Supporting Information.

### 2.6 ROS imaging

For dihydroethidium (DHE) staining, in situ ROS detection was performed using DHE (Beyotime, S0063). Images were captured and analyzed using the Olympus FV 1200 imaging system. For details, please see Supporting Information.

### 2.7 Genomics DNA extraction and library construction

For each group, 8 samples of female flies were examined and each sample with 25 guts was respectively collected and sent to the BGI. The microbial community DNA was extracted using MagPure Stool DNA KF kit B. DNA was quantified and the quality was checked. Variable regions V3–V4 of bacterial 16S rRNA gene were amplified with degenerate PCR primers. For details, please see Supporting Information.

### 2.8 Statistical analysis

GraphPad Prism9 was used to perform the statistical analysis. PcoA and α‐diversity were performed by package vegan version 2.5‐7 of R version 4.1.2. For functional pathways, a phylogenetic investigation of communities by the reconstruction of unobserved states (PICRUSt2) algorithm was performed. For details, please see Supporting Information. All data are shown as means ± SEM.

## 3 RESULTS

### 3.1 DNA damage induces high ROS levels

**Figure 1:**
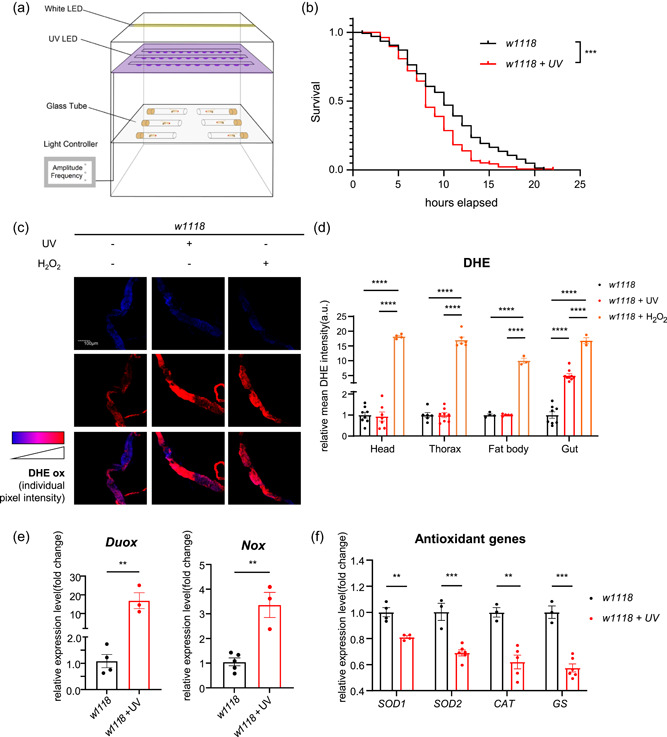
DNA damage induces reactive oxygen species (ROS) accumulation in *w1118* flies. (a) Schematic of the experimental setup. (b) Oxidative stress curves of *w1118* female flies. *n* > 120, log‐rank (Mantel–Cox) test. (c) Oxidized DHE (DHEox) reports ROS in the gut. (d) Quantification of DHEox in the head, thorax, fat body, and gut. *n*≥ 3, one‐way ANOVA with Bonferroni post hoc test. (e) mRNA levels of *Duox* and *Nox* in the gut. *n* ≥ 3, unpaired *t*‐test. (f) mRNA levels of *SOD1*, *SOD2*, *CAT*, and *GS* in the gut. *n* ≥ 3, unpaired *t*‐test. All data shown as mean ± SEM, **p < 0.01, ***p < 0.001, ****p < 0.0001. DHE, dihydroethidium.

Narrow‐band UV LEDs generate DNA damage and broad‐band white LEDs simulate alternating light‐dark conditions for fly rearing (Figure 1a). To examine the viability of health after DNA damage, we examined the survival under oxidative stress of *w1118* flies. Both female and male flies revealed worse survival under oxidative stress after DNA damage (Figure 1b and Supporting Information:  Figure S1). For the ROS levels, the guts of exposed flies showed an increase (Figure 1c) rather than other tissues which appeared indistinguishable (Figure 1d and Supporting Information: Figure S2). The H_2_O_2_‐treated group served as a positive control. The expression of *Duox* and *Nox* significantly increased in DNA‐damage groups (Figure 1e), while the antioxidant genes including *superoxide dismutase 1* (*SOD1*), *superoxide dismutase 2* (*SOD2*), *catalase* (*CAT*), and *glutathione synthetase* (*GS*) significantly decreased (Figure 1f). These data suggested that DNA damage contributed to ROS accumulation in the gut tissue.

### 3.2 Parp1 is activated after DNA damage

**Figure 2:**
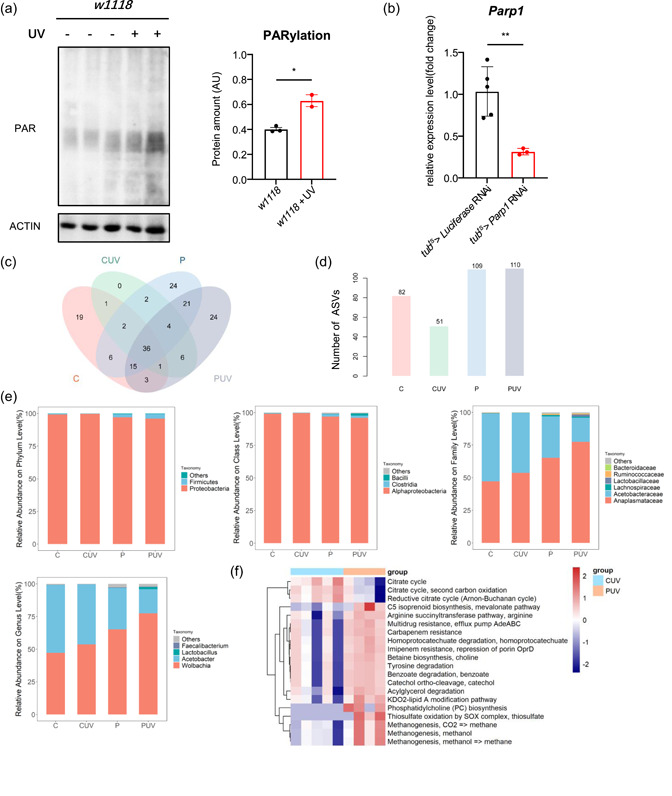
Microbiome and function analysis for Parp1 knockdown flies under DNA damage. (a) PARylation levels and quantification in the whole body of *w1118* flies. *n* ≥ 2, unpaired *t*‐test. (b) mRNA levels of *Parp1* of *tub‐gal4* ^*ts*^ flies. *n* ≥ 3, unpaired *t*‐test. (c–f) Microbiome analysis of 16S rDNA sequencing data from *tub‐gal4* ^*ts*^ > *Luciferase* RNAi (C), *tub‐gal4* ^*ts*^ > *Parp1* RNAi (P), and UV exposed groups, respectively, abbreviated as (CUV) and (PUV). (c) Prediction of species composition level similarity with a Venn diagram. (d) The ASVs of samples from four groups. (e) Microbiome analysis was performed at phylum, class, family, and genus levels. (f) Relative abundance of KEGG pathways of functional categories in the gut microbiota. All data shown as mean ± SEM, *p < 0.05, **p < 0.01.

DNA damage is the reason for the activation of PARP1. PARylation was enhanced after exposure in *w1118* flies (Figure 2a). To investigate the role of PARP1 in DNA damage, we generated Parp1 global knockdown flies with the tub‐gal4; tub‐gal80ts (*tub‐gal4* ^*ts*^) driver. The mRNA levels of *Parp1* decreased in flies with Parp1 RNAi (Figure 2b).

### 3.3 DNA damage alters the intestine microbiome under Parp1 knockdown

Intestinal homeostasis is represented by the direct variations in the host commensal community. DNA damaged flies were examined by 16S rDNA sequencing. One domain, 1 kingdom, 9 phyla, 14 classes, 28 orders, 51 families, 103 geneses, and 163 species were detected. The *tub‐gal4^ts^>Luciferase* RNAi flies (Group C) served as the control. The results of α diversity analysis showed that DNA damage affected negatively the diversity of the microbiome by comparing the Group C with exposed *tub‐gal4* ^*ts*^ > *Luciferase* RNAi flies (Group CUV; Supporting Information: Figure S3A). The *tub‐gal4* ^*ts*^ > *Parp1* RNAi flies (Group P) and exposed flies (Group PUV) showed an increase in the α diversity analysis by comparing with the Group CUV. The Venn diagram of ASVs (Figure 2c) and the species abundance of bacteria (Figure 2d) showed differences in microorganisms and the total number of species between the four groups.

DNA damage and Parp1 inhibition played independent and synergistic effects on the relative abundance of species at each classification level of the gut microbiome. At the phylum level, DNA damage promoted the abundance of *Proteobacteria*, similar to patients with intestinal immune diseases (Frank et al., 2007). Oppositely, Parp1 knockdown reduced the abundance of *Proteobacteria*. The abundance of *Firmicutes* increased in Group PUV, similar to Yanomami people who lived under high sunlight damage (Conteville & Vicente, 2020). At the class level, DNA damage promoted the abundance of *Alphaproteobacteria*, which belongs to the Proteobacteria phylum. At the family level, DNA damage promoted the abundance of Anaplasmataceae and reduce Lachnospiraceae, Acetobacteraceae, and Ruminococcaceae. Similarly, Parp1 knockdown promoted the abundance of Anaplasmataceae and reduce Acetobacteraceae. Oppositely, Lachnospiraceae and Ruminococcaceae increased in Groups P and PUV. At the genus level, DNA damage promoted the abundance of *Wolbachia*, while reducing the abundance of *Acetobacter* which is similar to Parp1 knockdown flies (Figure 2f). The effect of DNA damage in Parp1 knockdown flies showed a different pattern. At the phylum level, DNA damage promoted the abundance of *Firmicutes*. At the class level, DNA damage promoted the abundance of *Bacilli*. At the family level, DNA damage promoted the abundance of Lactobacillaceae and Anaplasmataceae. At the genus level, DNA damage promoted the abundance of *Lactobacillus* (Figure 2e).

### 3.4 DNA damage alters the microbial function under Parp1 knockdown

The functional prediction results showed that the COG functional composition of the four groups was different (Supporting Information: Figure S3B). Group PUV flies revealed the least functional abundance in amino acid transport and metabolism, inorganic ion transport, and metabolism but the highest abundance in replication, recombination, and repair together with translation, ribosomal structure, and biogenesis. Functional differences were performed between CUV and PUV based on Kyoto Encyclopedia of Genes and Genomes (KEGG) annotations, indicating that 20 KEGG pathways were significantly altered in the gut microbiota (Figure 2f). Compared with control, Parp1 knockdown flies revealed less abundance in microbiotas related to the citrate cycle, second carbon oxidation‐related pathways, and reductive citrate cycle (Arnon‐Buchanan cycle), leading to variations in energy production. On the contrary, the Parp1 knockdown flies revealed a higher abundance of phosphatidylcholine biosynthesis, thiosulfate oxidation, and methanogenesis, associated with the production of methane and microbial metabolism.

### 3.5 Parp1‐specific knockdown exacerbates ROS

**Figure 3:**
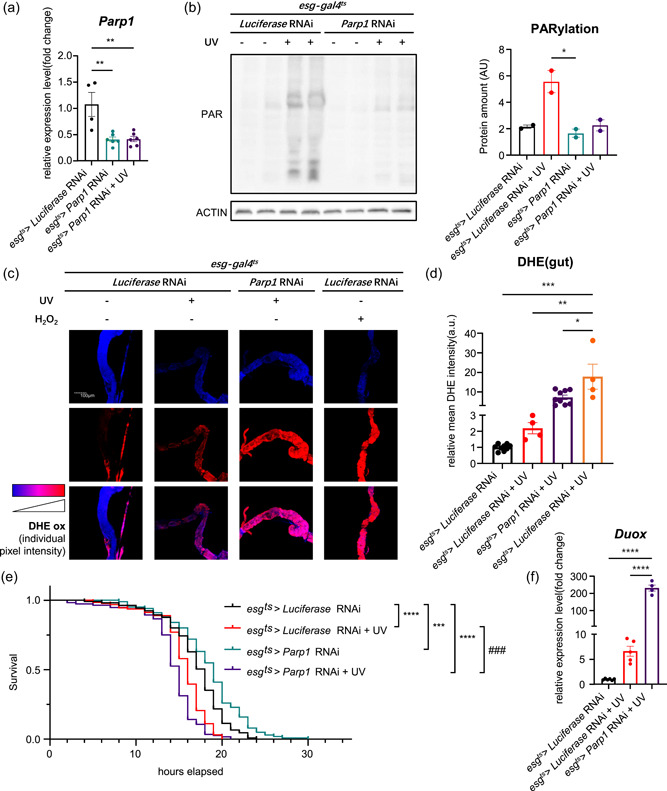
Parp1‐specific knockdown causes reactive oxygen species (ROS) accumulation under DNA damage. (a) mRNA levels of *Parp1* in gPARPKD flies. *n* ≥ 4, one‐way analysis of variance (ANOVA) with Bonferroni post hoc test. (b) PARylation levels and quantification in the gut of gPARPKD flies. *n* = 2, one‐way ANOVA with Bonferroni post hoc test. (c) DHEox of ROS in the gut of gPARPKD flies. (d) Quantification of DHEox. *n*≥ 4, one‐way ANOVA with Bonferroni post hoc test. (e) Oxidative stress curves of gPARPKD flies with UV. *n* ≥ 100, log‐rank (Mantel–Cox) test. (f) mRNA levels of *Duox* in the gut of gPARPKD flies. *n* ≥ 4, one‐way ANOVA with Bonferroni post hoc test. All data shown as mean ± SEM, *p < 0.05, **p < 0.01, ***p < 0.001, ****p < 0.0001. # denotes differences between the *esg‐gal4* ^*ts*^ > *Luciferase* RNAi + UV groups.

To refine the tissue‐specific physiology, we used intestinal stem cells and enteroblasts (*esg‐gal4* ^*ts*^) driver to knock down Parp1 expression specifically in the gut (gPARPKD) and the mRNA levels of *Parp1* decreased (Figure 3a). PARylation enhanced in the gut tissue after DNA damage and decreased in Parp1 RNAi groups (Figure 3b). High ROS levels were observed in exposed *esg‐gal4* ^*ts*^ > *Parp1* RNAi flies (Figure 3c,d). H_2_O_2_ also served as a positive condition. The viability of health showed that *esg‐gal4* ^*ts*^ > *Parp1* RNAi flies with additional DNA damage revealed the worst survival under oxidative stress (Figure 3e). The expression of *Duox* significantly increased by up to 200‐fold for *esg‐gal4* ^*ts*^ > *Parp1* RNAi after DNA damage (Figure 3f). However, the expression of *Nox* showed no significant difference following the Parp1 knockdown (Supporting Information: Figure S4A). Antioxidant genes, including *SOD1*, *SOD2*, *CAT*, and *GS*, significantly decreased in gPARPKD flies with DNA damage (Supporting Information: Figure S4B). These data suggested that Parp1 inhibition and DNA damage additively contribute to ROS accumulation in the gut tissue and eventually become harmful to the individual vitality.

### 3.6 DNA damage induces suppression in AMPs

Intestinal homeostasis is achieved through ROS and other several strategies (Guo et al., 2014; Vesala et al., 2020): the production of AMPs, the melanization reaction, and phagocytosis. DNA damage did not exert enhancement on the melanization reaction and phagocytosis. The expression of phagocytic receptors and opsonin in the gut of exposed *w1118* flies, including the scavenger receptor *Croquemort (Crq)*, *Draper (Drpr), integrin betanu subunit (Itgbn)*, *thioester‐containing protein (Tep2* and *Tep4)*, and *Peste* showed no significant difference (Supporting Information: Figure S5A). Similarly, the expression of negative regulator *Srpn27* and positive regulator *MP1* for melanization reaction also showed no significant difference after DNA damage (Supporting Information: Figure S5B).

**Figure 4:**
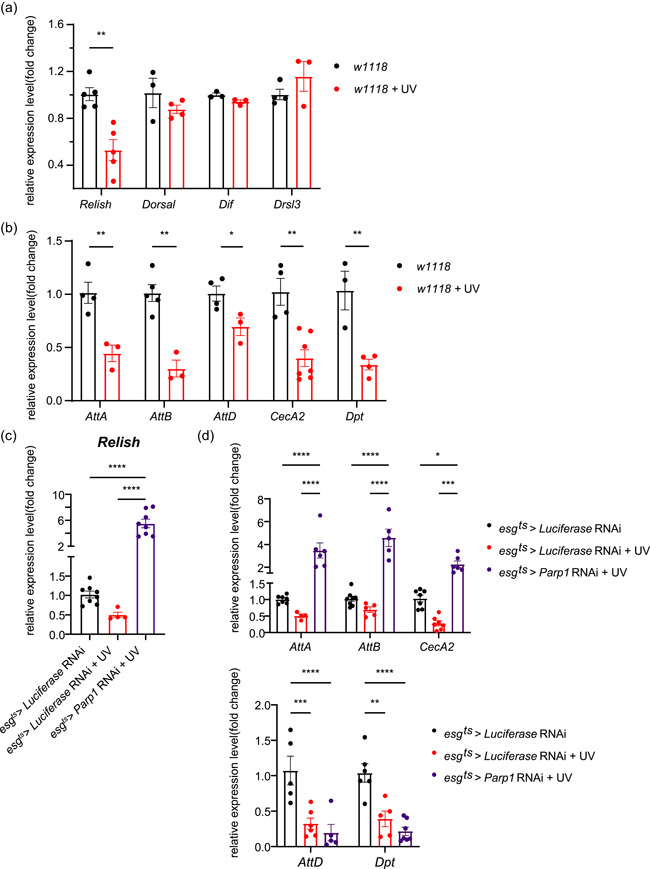
Parp1 inhibition promotes the secretion of AMPs. (a) mRNA levels of *Relish*, *Dorsal*, *Dif*, and *Drsl3* in the gut of *w1118* flies, *n* ≥ 3. (b) mRNA levels of *AttA*, *AttB*, *AttD*, *CecA2*, and *Dpt* in the gut of *w1118* flies, *n* ≥ 3. (c) mRNA levels of *Relish* in the gut of gPARPKD flies, *n* ≥ 4. (d) mRNA levels of *AttA*, *AttB*, *CecA2*, *AttD*, and *Dpt* in the gut of gPARPKD flies, *n* ≥ 4. All data shown as mean ± SEM. For two‐group comparisons: unpaired *t*‐test. For more than two groups: one‐way analysis of variance with Bonferroni post hoc test. *p < 0.05, **p < 0.01, ***p < 0.001, ****p < 0.0001.

However, DNA damage caused suppression in IMD/Relish‐targeted AMPs. AMPs are secreted by Toll and IMD of NF‐kB signaling or JAK‐STAT pathway in *Drosophila* (Buchon et al., 2009). The expression of genes encoding NF‐κB subunits (*Relish* for IMD pathway, *Dorsal*, and *Dif* for Toll pathway) showed that DNA damage reduced the level of *Relish* (Figure 4a). The expression of JAK–STAT target, *drosomycin‐like 3*(*drsl3*), also showed no significant difference after DNA damage (Figure 4a). Additionally, the expression of NF‐κB targets including *Attacin A* (*AttA*), *Attacin B* (*AttB*), *Attacin D* (*AttD*), *CecropinA2* (*CecA2*), and *Diptericin* (*Dpt*) also decreased following DNA damage (Figure 4b).

Collectively, these data suggested that under DNA damage, the host defense is achieved neither by the inactivated melanization reaction, phagocytosis or by the inhibited AMPs. Accumulation of ROS played a dominant role.

### 3.7 Parp1‐specific knockdown contributes to AMP‐secretion through IMD/Rel

The production of AMPs after DNA damage in gPARPKD flies was examined. Similar to *w1118* flies, there was a reduction in the *Relish* expression of *esg‐gal4* ^*ts*^ > *luciferase* RNAi flies after DNA damage, while Parp1 knockdown resulted in increased expression of *Relish* in gPARPKD flies (Figure 4c). The expression of Relish‐targeted AMPs showed that *AttA*, *AttB*, and *CecA2* increased in the Group *esg‐gal4* ^*ts*^ > *Parp1* RNAi after DNA damage, while *AttD* and *Dpt* remained inhibited (Figure 4d).

**Figure 5:**
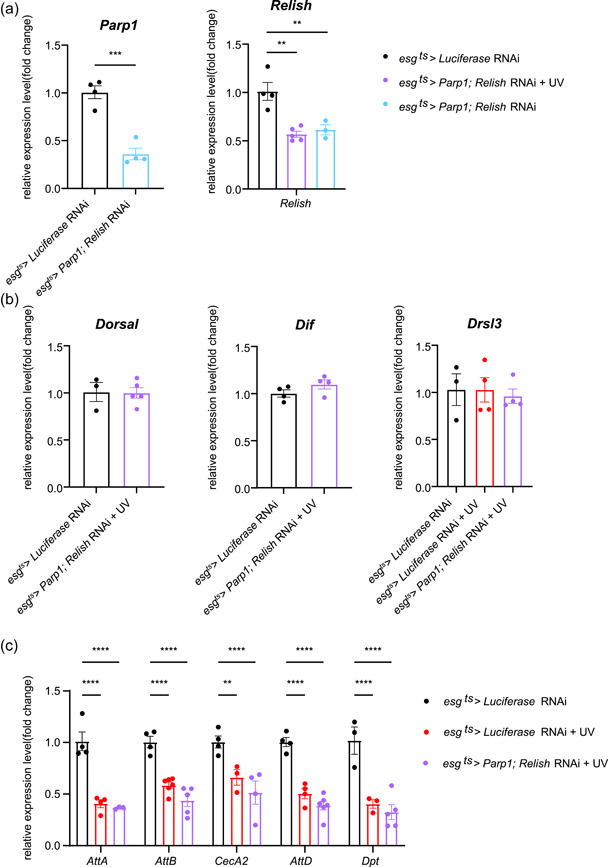
Relish is required for AMP secretion. (a) mRNA levels of *Parp1* and *Relish* in the gut of gDKD flies, *n* ≥ 3. (b) mRNA levels of *Dorsal*, *Dif*, and *Drsl3* in the gut of gDKD flies, *n* ≥ 3. (c) mRNA levels of *AttA*, *AttB*, *CecA2*, *AttD*, and *Dpt* in the gut of gDKD flies, *n* ≥ 3. All data are shown as mean ± SEM. For two‐group comparisons: unpaired *t*‐test. For more than two groups: one‐way analysis of variance with Bonferroni post hoc test. **p < 0.01, ***p < 0.001, ****p < 0.0001.

To verify the role of Relish in mediating the function of Parp1, we generated Relish and Parp1 double‐specific knockdown in the gut of flies (gDKD). The mRNA levels of *Parp1* and *Relish* decreased in gDKD flies (Figure 5a). By examining the expression of genes encoding other responses we found the expression of other NF‐κB subunits (*Dorsal* and *Dif*; Figure 5b) and the target of the JAK‐STAT pathway (*Drsl3*) unsurprisingly showed no significant differences in gDKD flies after DNA damage (Figure 5b). For IMD/Relish targets, the expression of *AttA*, *AttB*, and *CecA2* decreased in the Group gDKD compared with gPARPKD flies (Figure 5c) after DNA damage, while the expression of *AttD* and *Dpt* kept decreasing in both exposed gPARPKD and gDKD flies (Figure 5c).

Collectively, these data suggested that Parp1 knockdown exacerbates the host defense responses for DNA damage through IMD/Rel signaling. At the same time, both the Toll and JAK‐STAT pathway did not play compensatory roles.

## 4 DISCUSSION

Besides its well‐known application in the genetic region, *Drosophila* has emerged as a tool in studies for ROS, given that flies share the majority of mitochondrial and energy metabolic pathways with humans for the source of ROS generation. The simplicity in anatomy enables *Drosophila* to be employed in ROS studies and provides an immediate strategy for ROS quantification through unfixed staining (Vaccaro et al., 2020). By counting the death events, *Drosophila* offers an advantage over mammals in quantifying endogenous oxidative stress and vitality.

Biologically relevant doses of UV generate ROS in vitro (O'Donovan et al., 2005). Under DNA damage, we observed ROS accumulation in the gut tissue rather than other organs, which leads us to focus on the gut tissue in this study. Individual homeostasis is achieved through several strategies (Guo et al., 2014; Li et al., 2020): ROS, the production of AMPs, the melanization reaction, and phagocytosis. Usually, ROS serves as a first line of defense while AMPs and other strategies of defense act as eliminating antioxidant pathogens. Studies have focused on the whole body while the reaction of the gut has been masked (Buchon et al., 2009). ROS is a byproduct of mitochondrial energy metabolism and is generated under DNA damage (Finkel & Holbrook, 2000). A significant fraction of ROS is made through the action of two conserved enzymes, Nox and Duox (Ha et al., 2009). Apart from the benefits in host defense, overproduction of ROS would lead to another story. The dynamic cycle of ROS generation and elimination is vital. UV contributes to the reduction of antioxidant genes. In human melanocytes and dermal fibroblasts, UV‐B reduced the total and basal antioxidant GSH content (Shin et al., 2018; Upadhyay et al., 2022). Also, the activity of another antioxidant enzyme, catalase, was reduced to UV light in the model organism (Zhang et al., 2020). The imbalance between the generation and endogenous antioxidant systems in the gut causes ROS accumulation and could eventually be fatal (Ha, Oh, Ryu, et al., 2005).

The intestinal commensal community of *Drosophila*, which shares a large part of overlap with humans, represents intestinal homeostasis and has been employed to better define the relationship of microbiota with host defense and the molecular basis of pathophysiological traits. We observed independent and synergistic effects of DNA damage and Parp1 inhibition on gut microbiome and metabolism. The α‐diversity increased in Parp1 knockdown flies while the microbial functional predictions showed downregulation of metabolic pathways related to the citrate cycle following DNA damage. Patients with intestinal immune diseases are characterized by the overgrowth of potentially pathogenic bacteria, an increase in the abundance of *Proteobacteria* (Frank et al., 2007), which has also been observed in flies after DNA damage. The human cohort study showed that environmental stress of UV would significantly increase the relative abundance of *Firmicutes* (Bosman et al., 2019), which has also been observed in the exposed Parp1 knockdown flies. The analysis of microbiota provides a better understanding of the accumulation of ROS. Expression of Duox rather than Nox showed a significant increase in exposed Parp1 knockdown flies. In accordance with the expression of NADPH enzyme, species of microbiota for the generation of Nox revealed no significant changes, such as *Lactobacillus* spp., *Akkermansia* spp. (Iatsenko et al., 2018). Pathogens rather than symbiotic bacteria of the commensal community contribute to the generation of Duox (Kim & Lee, 2014). The activation of AMPs for the defense of pathogens and the upregulation of Duox sense the variation in homeostasis and response in exposed Parp1 knockdown flies. In line with the functional conservation of the human Parp1 gene in flies, we show that Parp1 is activated under DNA damage through PARylation. PARP1 functions in an antagonistic way, which is like a longevity assurance factor through higher mitochondrial content in physiological conditions (Piskunova et al., 2008) and an aging‐promoting factor through apoptosis promotion in pathophysiological conditions (T. Liu et al., 2021; Mangerich & Bürkle, 2012). Parp1 inhibition reduces the level of PARylation countering DNA damage together with the accumulation of ROS to a harmful level (Chevanne et al., 2007). An increase in the expression of Duox represents the hazard of pathogens and leads to the activation of IMD/Relish targeted AMPs, which eventually change the responses of host defenses under DNA damage.

In conclusion, our data reinforce the evidence that DNA damage results in ROS accumulation while AMP secretion for host defense is suppressed. Microbiome and functional analysis also provide evidence for the hazard of DNA damage. Furthermore, the knockdown of PARP1 reduces the generation of PARylation, exacerbates ROS accumulation to a harmful level, and provokes the AMPs. These data suggest the notion that PARP1 inhibition changes the balance of host defense from ROS to IMD/Relish pathway AMPs under DNA damage specifically in the gut tissue.

## AUTHOR CONTRIBUTIONS

Xingxing Kong and Tiemin Liu designed the experimental plan. Yixiao Zhuang performed the majority of phenotypes in vivo and molecular experiments. Shanshan Guo performed molecular experiments. Shuang Zhang, Xiaorui Xing, Jiyang Ding, Xiaoyu Liu, Shuai Zhang, Xinyi Zhang, and Muyang He collected the data. Hui Wang performed the data analysis. Keneilwe Kenny Kaudimba participated in the article modification. Yixiao Zhuang wrote the manuscript incorporating edits and comments from Xingxing Kong, Tiemin Liu, and Li Jin.

## CONFLICT OF INTEREST

The authors declare no conflict of interest.

## Supporting information

Supporting information.

Supporting information.

Supporting information.
