# Supplementary material for: PARP1 inhibition enhances reactive oxygen species on gut microbiota
Source: J Cell Physiol. 2022 Aug 22;237(11):4169–79. doi: 10.1002/jcp.30861 (PMC9805012; doi:10.1002/jcp.30861)
Supplement: Supplementary file 1 — Supporting information. [file JCP-237-4169-s001.docx]

## *Fly strains and rearing*

The following fly strain was obtained from Bloomington Stock Center: *UAS-Relish* ^RNAi^ (RRID: BDSC_33661). *UAS-CG40411 Parp1* ^RNAi^ (II) (TH201500671.S, RRID: BDSC_57265) was obtained from Tsinghua Fly Center. *UAS-Luciferase* ^RNAi^ (RRID: BDSC_35788), *esg-gal4^ts^* (RRID: BDSC_92832), *tub-gal4^ts^* and wild type fly *w1118* (RRID: BDSC_3605) were gifted from Xinhua Lin (Fudan University). *Parp1* ^RNAi^*-Relish* ^RNAi^ double transgenic fly line was generated by standard recombination genetic crosses of *Parp1* ^RNAi^ and *Relish* ^RNAi^ lines. The used balancers and genetic markers are described in detail on FlyBase (<http://flybase.bio.indiana.edu/>) (Mascolo et al., 2022). For details, please seeing supplementary materials.

Flies were reared in standard vials (25 mm × 95 mm) containing cornmeal medium (1% agar, 2.45% yeast, 6.75% sucrose, 6.25% cornmeal, 1% propionic acid, and 1.75% methylparaben, all concentrations given in wt/vol).

*w1118* is a type of wild type fly, which was kept at 25℃. *w1118* flies were exposed to UV on day 30 when they have completely developed and were still energetic.

*Tub-gal4^ts^＞Luciferase* ^RNAi^*, tub-gal4^ts^＞Parp1* ^RNAi^*, esg-gal4^ts^＞Luciferase* ^RNAi^*, esg-gal4^ts^＞Parp1* ^RNAi^*, esg-gal4^ts^＞Parp1* ^RNAi^*; Relish* ^RNAi^ files were driven with tubgal80ts system and kept at 29℃ since the second day of the adult stage in a humidified, temperature-controlled incubator. For flies driven with tubgal80ts system, female flies were used in experiments and exposed to UV at 16-day old and dissected immediately after exposure.

Tubgal80ts system was used in transgenetic flies and these flies were exposed and examined on day 16. 16-day-old flies have got stable gut microbiome composition (1). The knockout of Parp1 during early stages could be lethal (2). To avoid the influence on development, we knock down the genes at the adult stage by tubgal80ts, which served as a temperature-sensitive controller. Specifically, the flies were placed at 29°C on the second day of adult stage for the genes to be knockdown. However, 29°C is not the most suitable temperature for living and these flies would grow twice as fast as in 25°C. To make sure that all the flies used in this study are under the same stage, we used 16-day-old flies with tubgal80ts at 29°C and 30-day-old *w1118* flies at 25°C.

Female flies are usually used in the study of gut tissue in flies (3-5). They have thick and large intestines compared with male flies. Phenotype studies in both genders (Figure 1B and Figure S1) have shown that the male and female flies reacted to UV in a similar pattern. Hence, female flies were used in the following study.

## *Oxidative stress resistance assay*

For oxidative stress resistance assay, 20 flies were first starved in a vial filled with 5 ml of 1% agar which served as water source. After starvation, flies were transferred into a vial with filter papers impregnated with 300μl of 5% H_2_O_2_ solution. The activity data were extracted at 1h bin by counting the number of dead flies. For each line, over 5 duplicates were made and in total, over 100 flies were typically used (38).

## *UV radiation*

Briefly, 20-25 flies were housed in each quartz glass vail, through which UVB irradiation was able to transmit. Flies were incubated in glass vials 1 day before the start of exposure. During the UV irradiation, flies were exposed to 0.6 mW/cm^2^ of UVB (peak emission of 313 nm) for 2 hours (6-9). The intensity of UVB irradiation was measured by a UVB detector (PMA2106-LLH, SOLAR LIGHT CO., INC.).

UVB exposure of 0.6 mW/cm^2^ was applied for 2 hours in this study. The intensity has been proved effective for adult flies (6, 7). Flies would adapt to the environmental UV light in 1 hour (8, 9). Long time exposure would be lethal for flies. 2-hour duration was applied which was both effective for the study and adequate for survival.

## *Protein extraction and Western Blot*

The protein extraction and western blot were carried out as described in the previous study (43). 25-30 flies were used in each biological replicate. The whole body or gut of *drosophila* was collected and extracted using RIPA buffer (Beyotime, P0013B) with protease inhibitor cocktail (Beyotime, P1005) and phosphatase inhibitor cocktail (Beyotime, P1091). The samples (50 ugs) were loaded onto SDS-PAGE under standard conditions, transferred, and probed with the antibodies.

## *Quantitative real-time PCR*

The quantitative real-time PCR was carried out as described in the previous study (44, 45). RNA was extracted using TRIzol reagent (Invitrogen) according to standard protocol. The cDNA was converted using a High-Capacity cDNA Reverse Transcription kit (AB applied biosystems, 4368813). Quantitative real-time PCR (qPCR) was performed on QuantStudis (2014-life-Q7) using the QuantiTect SYBR Green PCR kit (QIAGEN, 208504). The standardized RP49 mRNA was used as the invariant control. [Table S](#_Table_S1_sequence)1 lists the sequence of used primers in the study.

## *ROS imaging*

ROS are unstable and difficult to detect because of their high reactivity. For DHE staining, in situ ROS detection was performed using dihydroethidium (DHE) (Beyotime, S0063). Dihydroethidium (DHE) was used in this study which emitted blue fluorescence unless oxidized when it intercalated into DNA and fluoresced red (35). Flies were anesthetized on ice and dissected in PBS. Whole tissues were then incubated at 37°C with 5 μM DHE for 30 minutes in dark. Before mounting, samples were washed three times in PBS for 5 minutes at room temperature. Samples from each independent experiment were mounted between glass slides and coverslips. Images were captured and analyzed using Olympus FV 1200 imaging system.

## *Genomics DNA extraction*

For each group, 8 samples of female flies were examined and each sample with 25 guts was respectively collected and sent to the BGI (Shenzhen, China). The microbial community DNA was extracted using MagPure Stool DNA KF kit B (Magen, China) following the manufacturer’s instructions. DNA was quantified with a Qubit Fluorimeter by using a Qubit dsDNA BR Assay kit (Invitrogen, USA) and the quality was checked by running an aliquot on 1% agarose gel.

## *Library Construction*

Variable regions V3–V4 of bacterial 16S rRNA gene were amplified with degenerate PCR primers, 341F (5’-ACTCCTACGGGAGGCAGCAG-3’) and 806R (5’-GGACTACHVGGGTWTCTAAT-3’). Both forward and reverse primers were tagged with Illumina adapter, pad, and linker sequences. PCR enrichment was performed in a 50 μ L reaction containing 30ng template, fusion PCR primer, and PCR master mix. PCR cycling conditions were as follows: 94 ℃ for 3 minutes, 30 cycles of 94 ℃ for 30 seconds, 56 ℃ for 45 seconds, 72 ℃ for 45 seconds and final extension for 10 minutes at 72 ℃ for 10 minutes. The PCR products were purified with AmpureXP beads and eluted in the Elution buffer. Libraries were qualified by the Agilent 2100 bioanalyzer (Agilent, USA). The validated libraries were used for sequencing on the Illumina HiSeq2500 platform (BGI, Shenzhen, China) following the standard pipelines of Illumina, and generating 2 × 300 bp paired-end reads.

## *Statistical analysis*

GraphPad Prism9 was used to perform the statistical analysis. For two-group comparisons: either an unpaired t-test was used when data met the criteria for parametric analysis (normal distribution assessed by Shapiro-Wilk normality test using Prism9) or non-parametric analysis. For more than two groups comparison of mRNA qPCR results, ANOVA with Bonferroni post hoc test was performed. For comparison of survival curves, log-rank (Mantel-Cox) test was used. Principal coordinate analyses (PcoA) and Alpha diversity were performed by package vegan version 2.5-7 of R version 4.1.2. For functional pathways, a phylogenetic investigation of communities by the reconstruction of unobserved states (PICRUSt2) algorithm was performed. All data are shown as means ± SEM.

## *Reference*

1. Li H, Qi Y, Jasper H. Preventing Age-Related Decline of Gut Compartmentalization Limits Microbiota Dysbiosis and Extends Lifespan. Cell host & microbe. 2016;19(2):240-53.

2. Miwa M, Hanai S, Poltronieri P, Uchida M, Uchida K. Functional analysis of poly(ADP-ribose) polymerase in Drosophila melanogaster. Molecular and cellular biochemistry. 1999;193(1-2):103-7.

3. Kim B, Kanai MI, Oh Y, Kyung M, Kim EK, Jang IH, et al. Response of the microbiome-gut-brain axis in Drosophila to amino acid deficit. Nature. 2021;593(7860):570-4.

4. Schretter CE, Vielmetter J, Bartos I, Marka Z, Marka S, Argade S, et al. A gut microbial factor modulates locomotor behaviour in Drosophila. Nature. 2018;563(7731):402-6.

5. Iatsenko I, Boquete JP, Lemaitre B. Microbiota-Derived Lactate Activates Production of Reactive Oxygen Species by the Intestinal NADPH Oxidase Nox and Shortens Drosophila Lifespan. Immunity. 2018;49(5):929-42.e5.

6. Baik LS, Fogle KJ, Roberts L, Galschiodt AM, Chevez JA, Recinos Y, et al. CRYPTOCHROME mediates behavioral executive choice in response to UV light. Proc Natl Acad Sci U S A. 2017;114(4):776-81.

7. Guntur AR, Gu P, Takle K, Chen J, Xiang Y, Yang CH. Drosophila TRPA1 isoforms detect UV light via photochemical production of H2O2. Proc Natl Acad Sci U S A. 2015;112(42):E5753-61.

8. Fischbach KF. Simultaneous and successive colour contrast expressed in “slow” phototactic behaviour of walkingDrosophila melanogaster. Journal of comparative physiology. 1979;130(2):161-71.

9. Baik LS, Recinos Y, Chevez JA, Holmes TC. Circadian modulation of light-evoked avoidance/attraction behavior in Drosophila. PLoS One. 2018;13(8):e0201927.
