# Supplementary material for: PARP1 inhibition enhances reactive oxygen species on gut microbiota
Source: J Cell Physiol. 2022 Aug 22;237(11):4169–79. doi: 10.1002/jcp.30861 (PMC9805012; doi:10.1002/jcp.30861)
Supplement: Supplementary file 2 — Supporting information. [file JCP-237-4169-s002.docx]

## Supplementary Table 1 Sequence of primers

|  | Forward primer | Reverse primer |
| --- | --- | --- |
| *RP49* | GCAAGCCCAAGGGTATCGA | TAACCGATGTTGGGCATCAG |
| *Parp1* | CAAAATCCATGCCAGTATCACG | TCCAATTCGACCCCATGAAC |
| *Relish* | GACCCGAAAGCTCGGCGCAAA | TCGCTCACGAGTTGCGAGCAA |
| *Duox* | TGGCCAACGAGATAGTGATG | AAACTGCCATCAATCCAAGC |
| *Nox* | GGCTATCTCCTGCAAGATCG | CCAACTCAATCAGGCGGTAT |
| *AttA* | ACTCCCACATCAACGGACAT | GATGAGATAGACCCAGGCCA |
| *AttB* | GGCCCATGCCAATTTATTCA | CATTGCGCTGGAACTCGAA |
| *CecA2* | GGACAATCGGAAGCTGGTT | TGTGCTGACCAACACGTTC |
| *Dpt* | GTTCACCATTGCCGTCGCCTTAC | CCCAAGTGCTGTCCATATCCTCC |
| *AttD* | TCAGGCTTCAGGAAACCCAA | CTGGAGTGGAGGCGAATACT |
| *Drsl3* | TTGTCCTAATGGAGGCCAAC | GGCACTTTTCTCCACTCCAG |
| *SOD1* | CCACTGTGCTGATCTACTCTATTT | CTAACAGACCACAGGCTATGTATT |
| *SOD2* | GCGAAATAACGAGAACGTAAGC | TTACGGGCCACGAACATATC |
| *CAT* | GAATTCTCGACGCAGTCACA | CTGCAGCAGGATAGGTCCTC |
| *GS* | AGTTCACGGCCAATCTGTTC | ATCCTGACCACGATCCTCAC |
| *Crq* | GCGGGGAAACTCAACGAAAG | CTTGAGGGTAAGGCCATCCT |
| *Peste* | TCGCCAGCGGAATTTACCTC | ATGATCGTGAATTGGGGCTCA |
| *Drpr* | CTGGATGGACCCAATATCTGC | GTTTAATGCGATAGGTGGAGCA |
| *Itgbn* | GGAAGGGCGTTCTTGTGGATA | AGCGGATAAACATCTTTCGCAG |
| *Tep2* | ACAATGTCGTCGTCTCTGTCC | TGTACGAGGGTCCATTTAGACTG |
| *Tep4* | AGCCAGTCACCCTGAAAGTG | GCCGTCAGATTATATTCACCCG |
| *MP1* | GGATGCTATTGATGGGAACAAGC | CCCGGAGGTTTATGCAGGTG |
| *Srpn-27A* | TCTACCGCAAGACACTTAATTCC | CGGGATTGGTGAAGTCTAGCG |
