# Supplementary material for: PARP1 inhibition enhances reactive oxygen species on gut microbiota
Source: J Cell Physiol. 2022 Aug 22;237(11):4169–79. doi: 10.1002/jcp.30861 (PMC9805012; doi:10.1002/jcp.30861)
Supplement: Supplementary file 3 — Supporting information. [file JCP-237-4169-s003.pdf]

(A)

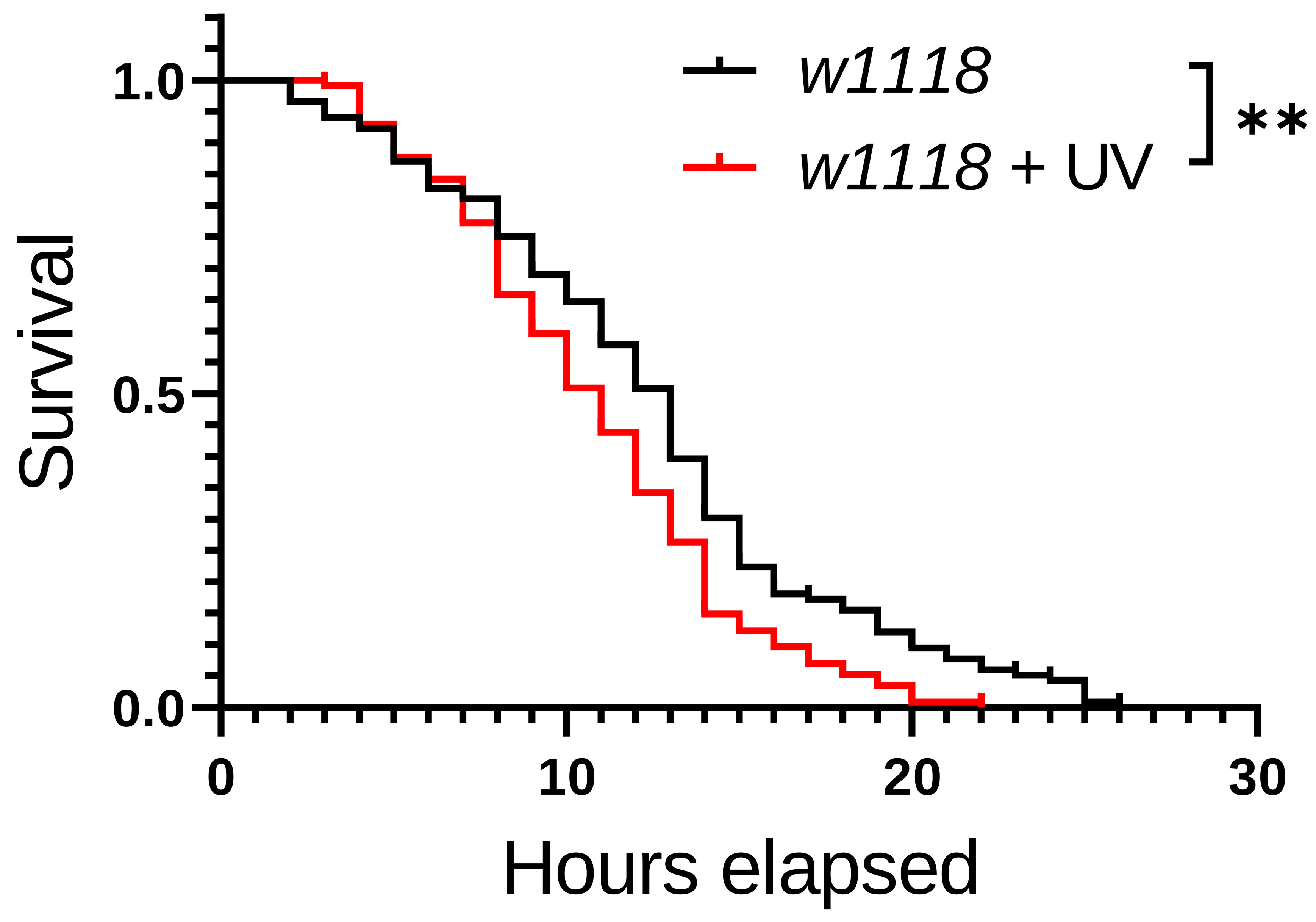

Supplementary Fig 1. UV exposure induces ROS in male *w1118* flies
(A) Oxidative stress curves of *w1118* male flies. n>100, log-rank (Mantel-Cox) test. **p < 0.01

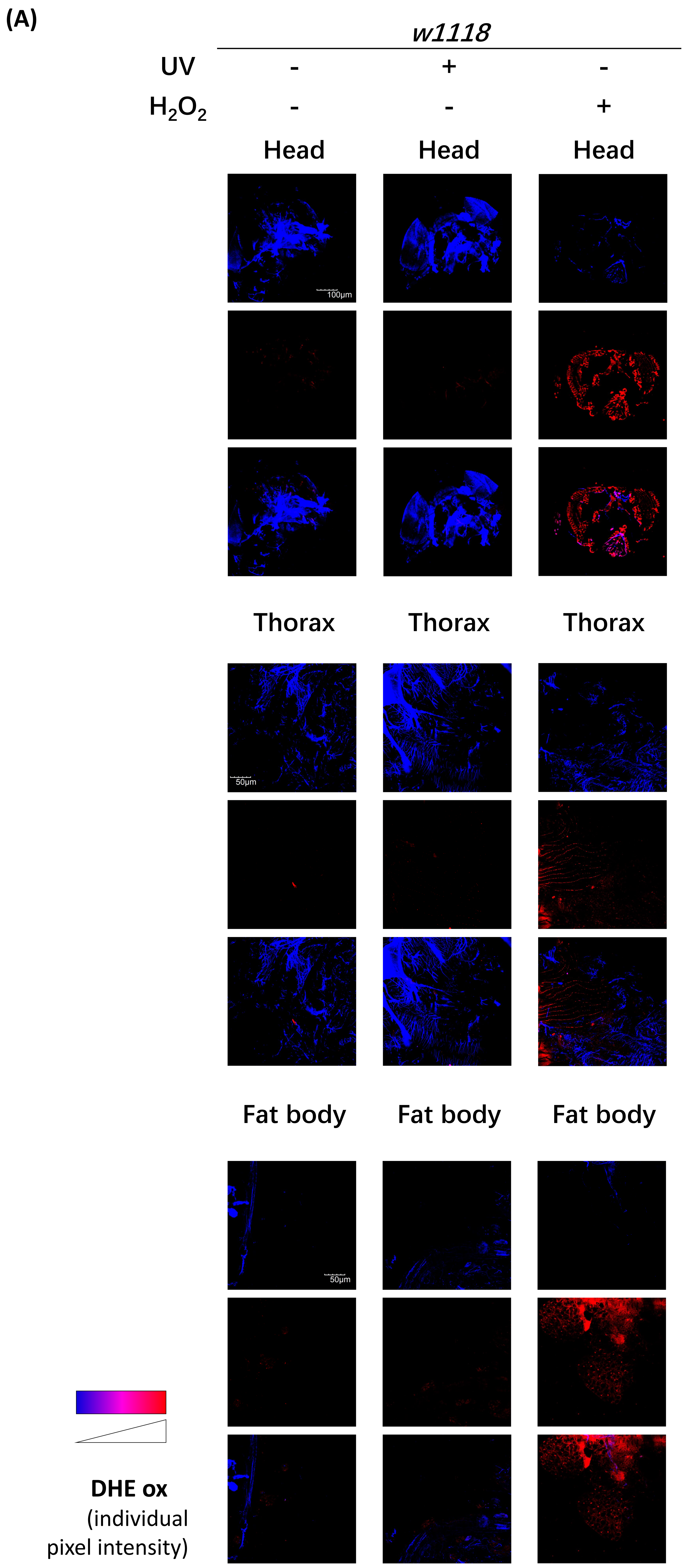

Supplementary Fig 2. DHE ox in *w1118* flies

(A) Oxidized DHE in the head, thorax and fat body of *w1118* flies.

(A)

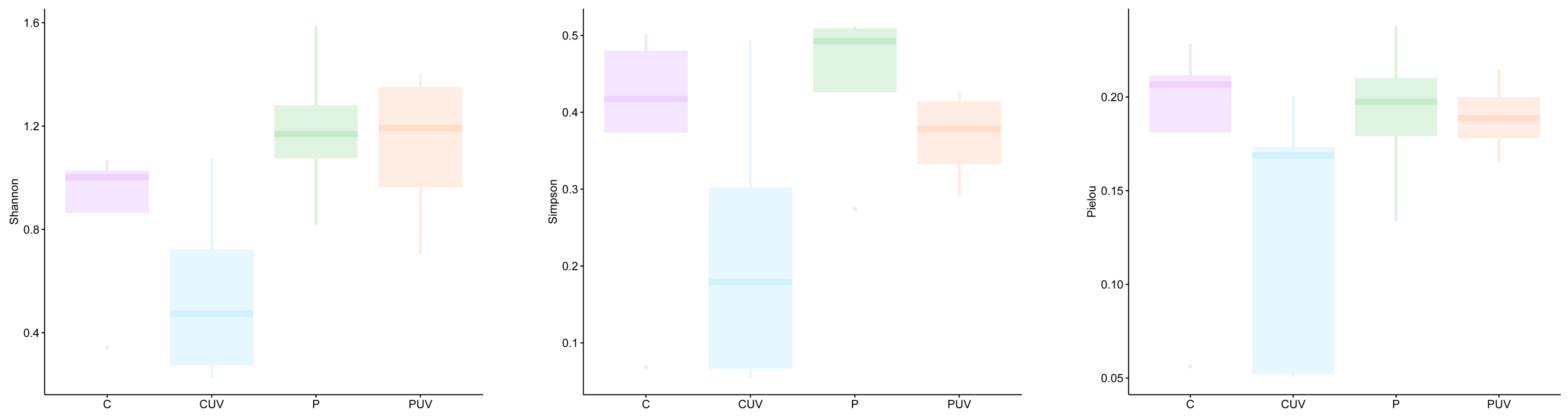

(B)

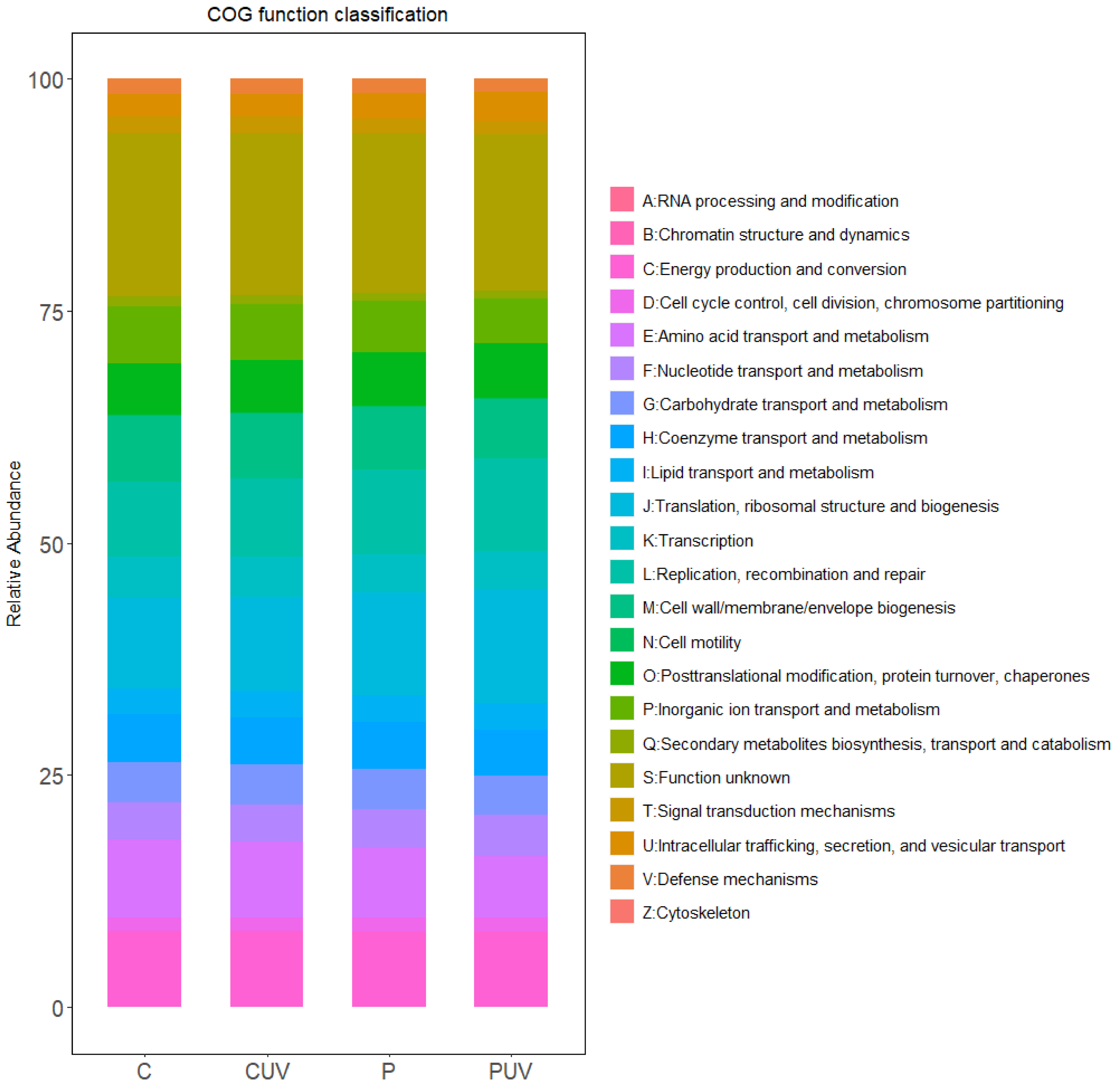

Supplementary Fig 3. Microbiome and function analysis for four groups
(A) α diversity indices statistics in four groups.
(B) COG function classification.

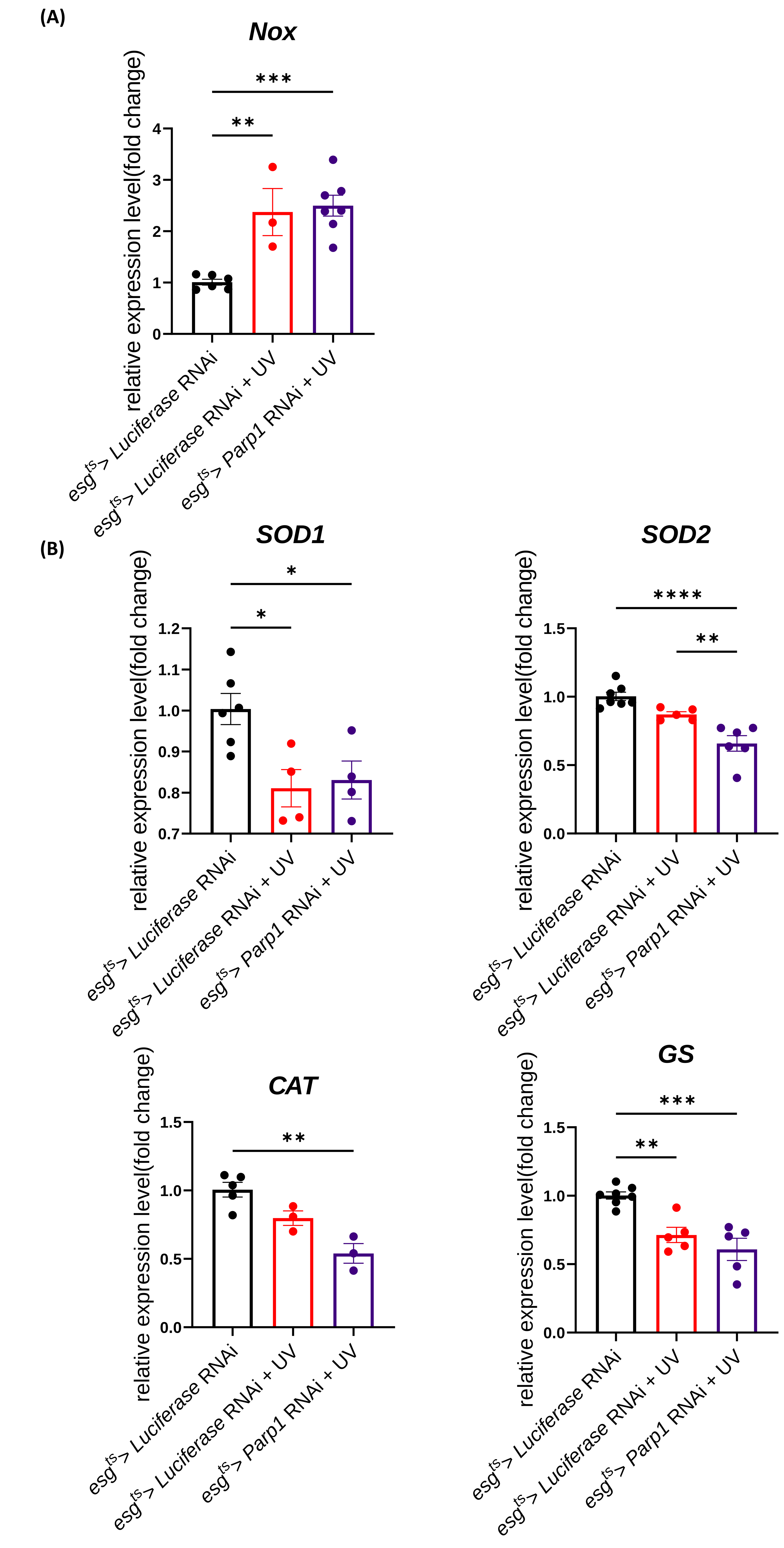

Supplementary Fig 4. Expression of NADPH enzyme and antioxidant genes
(A) mRNA levels of *Nox* in the gut of gPARPKD flies, n≥3.
(B) mRNA levels of *SOD1*, *SOD2*, *CAT* and *GS* in the gut of gPARPKD flies, n≥3.
All data shown as mean ± SEM, one-way ANOVA with Bonferroni post hoc test. $*p < 0.05$, $**p < 0.01$, $***p < 0.001$, $****p < 0.0001$.

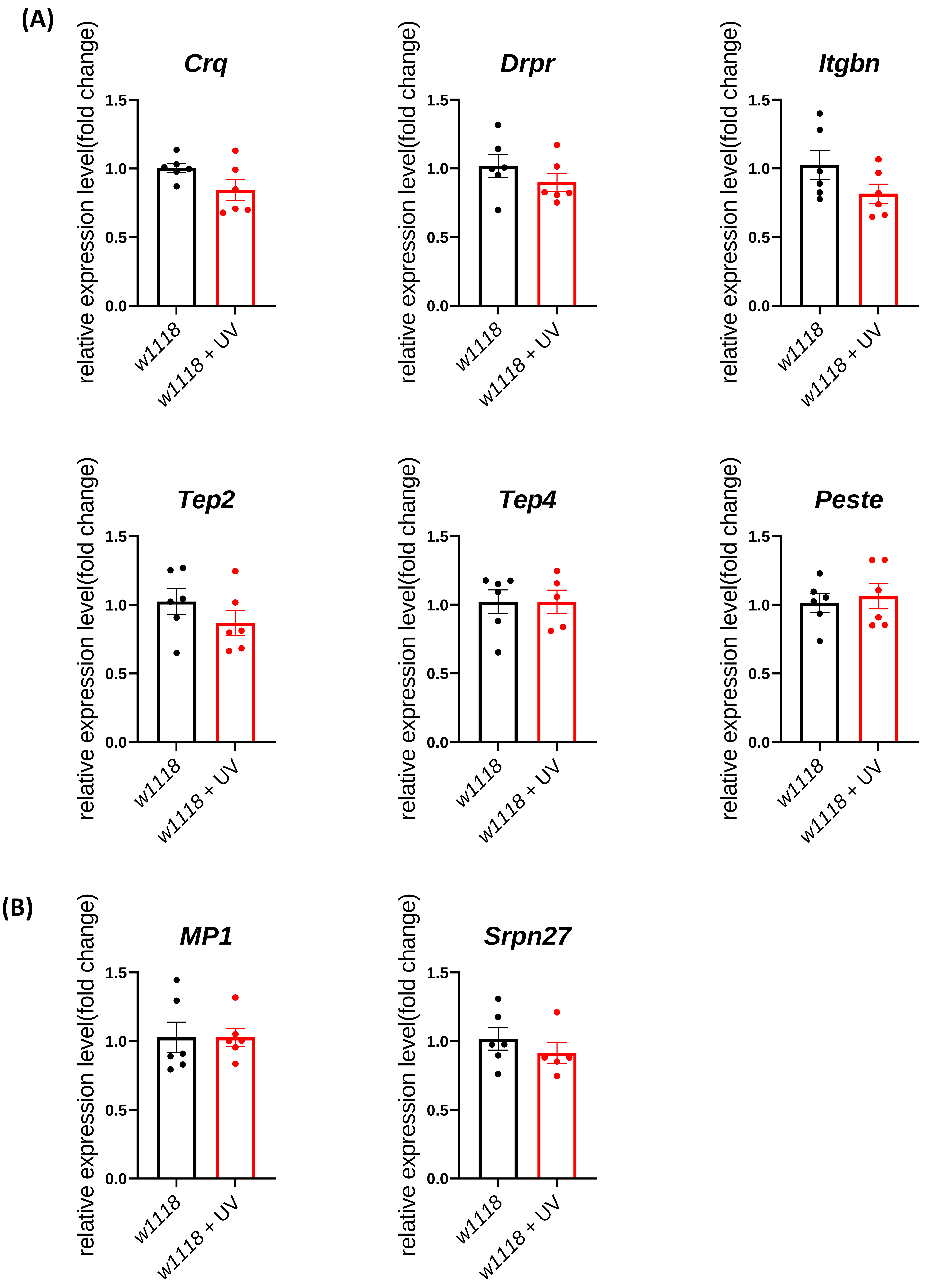

Supplementary Fig 5. Expression of phagocytosis, and melanization reaction related genes

(A) mRNA levels of *Crq*, *Drpr*, *Itgbn*, *Tep2*, *Tep4* and *Peste* in the gut of *w1118* flies, n≥5.

(B) mRNA levels of *MP1* and *Srpn27* in the gut of *w1118* flies, n≥5.

All data shown as mean ± SEM, unpaired t-test.
